# Evaluating large language models for abstract evaluation tasks: an empirical study

**DOI:** 10.3389/frma.2026.1807672

**Published:** 2026-05-04

**Authors:** Yinuo Liu, Emre Sezgin, Eric A. Youngstrom

**Affiliations:** 1 Institute for Mental and Behavioral Health Research, Nationwide Children's Hospital, Columbus, OH, United States; 2 Center for Biobehavioral Health, The Abigail Wexner Research Institute at Nationwide Children's Hospital, Columbus, OH, United States; 3 Department of Pediatrics, The Ohio State University College of Medicine, Columbus, OH, United States; 4 Department of Psychiatry and Behavioral Health, The Ohio State University, Columbus, OH, United States

**Keywords:** abstract evaluation, artificial intelligence, inter-rater reliability, large language models, peer-review

## Abstract

**Introduction:**

Large language models (LLMs) show great promise as tools for assisting scientific peer review, but their agreement with human experts in quantitative assessment of academic content needs further investigation. This study examined ChatGPT-5, Gemini-3-Pro, and Claude-Sonnet-4.5′s consistency and reliability in evaluating conference abstracts compared to one another and to human reviewers.

**Methods:**

Three LLMs independently graded 160 abstracts from a regional conference, while 14 human reviewers each assessed a subset using an identical rubric with eight criteria scored on a 1–5 scale. We compared AI and human scoring patterns using boxplots, calculated intraclass correlation coefficients (ICCs) for inter-rater reliability both among LLMs and between human and LLMs, and examined Bland-Altman plots to identify agreement patterns and systematic bias.

**Results:**

Three LLMs demonstrated high internal consistency with narrow interquartile ranges and few outliers in composite scores, while human reviewers exhibited greater scoring variability. LLMs also achieved good-to-excellent agreement with each other across all criteria (ICCs: 0.59–0.87). ChatGPT and Claude reached moderate agreement with human reviewers on overall quality and content-specific criteria, with ICCs = 0.45–0.60 for composite score, impression, clarity, objective, and results. The two LLMs' concordance with humans achieved fair levels on subjective dimensions, with ICC ranging from 0.23–0.38 for impact, engagement, and applicability. Gemini performed notably worse, showing fair agreement on half the criteria and poor reliability on impact and applicability. Bland-Altman analysis revealed acceptable or negligible systematic bias, with mean differences of 0.24 (ChatGPT), 0.42 (Gemini), and −0.02 (Claude) from human mean ratings.

**Discussion:**

With appropriate model selection, LLMs could reach moderate agreement with human experts on abstract overall quality and objective criteria, supporting their potential use for pre-screening low-quality submissions or serving as additional reviewers. Their ability to apply rubrics consistently across large volumes of abstracts offers advantages in efficiency and standardization that exceed human feasibility. However, LLMs' reduced performance on subjective dimensions indicates that they should complement rather than replace human judgment in abstract evaluation, with expert review remaining essential for comprehensive assessment.

## 1 Introduction

Peer review is fundamental to evaluations and selections of journals articles, grant applications, and conference presentations, yet this vital process faces two dominant issues: shortage in reviewers and concerns about rater agreement (Petrescu and Krishen, 2022). Reviewing is a time-consuming, voluntary task that requires scholars to spend hours reading material and writing feedback without compensation. Increasing submission volumes of manuscripts and proposals have strained the reviewer pool across scientific fields. As peer-review requests increase, scholars experience growing reviewer fatigue and decline more invitations (Breuning et al., 2015), leaving journals and conferences reporting greater difficulties at finding qualified evaluators (Fox et al., 2017). In addition to the recruitment pressures, judgement reliability has been a persistent issue in scientific review. We would hope that independent reviewers could agree on the content quality and provide similar ratings to the same subjects. However, most researchers have never received formal trainings in peer-review, and relevant resources or curriculum are either limited or difficult to access (Petrescu and Krishen, 2022; Willis et al., 2023). This lack of standardized trainings contributes to the lack of consensus in content quality evaluation. Studies have documented low-to-fair inter-rater reliability between reviewers for manuscripts and grant proposals (Bornmann and Daniel, 2010; Bornmann et al., 2010; Reinhart, 2009), raising concerns about inconsistent selection decisions and compromised validity of the peer-review process.

Abstract evaluation exemplifies both problems simultaneously. Reviewers face particularly intense workloads: they must evaluate dozens or hundreds of abstracts across diverse topics at conferences, and this number can increase to thousands for focused topics in systematic reviews (Needleman, 2002). The time-intensive nature of abstract review is magnified when multiple raters score each submission to generate aggregated evaluations, doubling or even tripling the resources required for decision-making. Reliability risks are even heighted for abstract evaluation. Abstracts provide substantially less information than full manuscripts or proposals (Rosmarakis et al., 2005), making consistent judgments inherently more difficult. Compounding this challenge, reviewers could be assigned abstracts outside their content expertise or evaluate only a subset of submissions rather than the full pool, both common practices in conferences that reduce consistency across reviewers (Deveugele and Silverman, 2017).

Artificial intelligence (AI) offers promising tools to help with these problems. AI technologies have already been applied across various stages of recruiting and selection processes, including writing job advertisements and screening applicant resumes (Gan et al., 2024; Hunkenschroer and Luetge, 2022). With the emergence of large language models (LLMs) (Naveed et al., 2025)—AI systems trained on text data that could understand and generate human-like languages—there is now a compelling opportunity to use AI to assist in or automate the evaluation of complex scientific material. By integrating LLMs into the evaluation workflow, the research community could alleviate the personnel pressure while devoting greater attention to reliability concerns underlying the traditional review process.

Previous studies have explored and validated AI's potential in academic reviewing. LLMs have showed reasonable accuracy at identifying scientific errors and verifying checklists, supporting their use as reviewing assistants (Liu and Shah, 2023). They could also play the role of reviewers and editors to accelerate the writing of constructive reports or decisions letters, reducing overall reviewing overload (Hosseini and Horbach, 2023). While LLMs' actual performance for complete evaluations of manuscripts or proposals needs further investigation, they show good promise in facilitating the peer-review process (Lee et al., 2025). Based on a large-scale randomized study in the International Conference on Learning Representations, many reviewers found the AI-generated feedback helpful and incorporated the suggestions into their reviews (Thakkar et al., 2026). In the specific context of abstract evaluation, LLMs have demonstrated strong performance in screening abstracts for systematic reviews and meta-analyses, delivering detailed assessment based on inclusion criteria or evaluating content against appraisal checklists (Zhou and Hu, 2025; Li et al., 2024). However, when LLM assessments are directly compared with human judgment, important limitations emerge. Shcherbiak et al. (2024) reported disappointingly low agreement between AI and human reviewers at scoring conference abstracts, raising questions about the reliability of AI-generated evaluations. Systematic investigation into when and how LLMs can reliably assess scientific material, particularly in high-stake contexts like conferences, is essential to establish the validity and reliability of integrating AI into the research evaluation workflow.

The present study contributes to this discussion through a comprehensive evaluation of LLM performance in abstract scoring. Using abstracts from a local research retreat, we compared three leading AI models—ChatGPT, Gemini, and Claude—across multiple dimensions: scoring consistency, inter-model agreement, and reliability with human expert judgment. This multi-model comparison provides empirical evidence about within-AI reliability and AI-human concordance in a real-world conference context.

## 2 Materials and methods

### 2.1 Sample

We used research abstracts (*N* = 160) from the Abigail Wexner Research Institute (AWRI) Research Retreat 2025 at Nationwide Children's Hospital as grading material. The abstracts were distributed across five research sessions: technology innovation (8.8%), pediatric research (37.5%), animal models (31.3%), intramural funding-related (15.0%), and general topics (43.8%); Table 1. Authors could choose multiple sessions, with 28.1% of abstracts assigned to more than one category. Each abstract begins with a title and a 50-word summary, followed by a structured abstract body (360-word limit) containing sections on importance, objective, methods, results, and conclusions.

**Table 1:** Research tracks and major focus.

Session topic	# of abstracts	Percentage
Technology innovation	14	8.8
Pediatric research	60	37.5
Animal models	50	31.3
Intramural funding related	24	15.0
General topic	70	43.8
Two or more sessions	45	28.1

### 2.2 Grading criteria

One judging rubric was used by human reviewers and LLM agents to evaluate all abstracts (Table 2). The rubric contained seven individual criteria, each rated on a 5-point Likert scale (1 = poor, 5 = excellent): impression, clarity, objective, results, impact, engagement, applicability. We also calculated the average of all criterion scores to define a composite score. Together, these eight component scores evaluated the abstract's overall quality (composite, impression), content-specific aspects (clarity, objective, results, impact), and broader relevance on audience interaction and applicability beyond its field (engagement, applicability).

**Table 2:** Judging rubric used by LLM and human reviewers.

Abbreviated name	Full criterion	Excellent (5)	Good (4)	Moderate (3)	Low (2)	Poor (1)
Impression	Overall impression	Abstract is compelling and reflects high-quality research	Strong overall impression, minor issues	Adequate, meets expectations	Below expectations, significant improvement needed	Does not meet academic or professional standards
Clarity	Clarity and organization	Abstract is well-structured, logical, and easy to follow	Mostly clear, minor issues in flow or organization	Adequate clarity but has some organizational issues	Difficult to follow, lacks coherent structure	Unclear and poorly organized
Objective	Research question/Objective	Clearly stated, focused, and significant	Clearly stated but may lack focus or significance	Adequately stated, somewhat vague or broad	Vague or not well explained	Not stated or irrelevant
Results	Results/Expected outcomes	Results are clearly described and support the research objective	Results are described but lack full context	Results or outcomes are vague or too briefly mentioned	Results are unclear or disconnected from objective	No results or expected outcomes presented
Impact	Significance/Impact	Clearly explains importance and potential impact of the work	Some explanation of significance, mostly relevant	Limited explanation of impact or relevance	Vague or weak justification for the work's importance	No discussion of significance
Engagement	Potential for engagement	High potential for an engaging, interactive, or thought-provoking presentation	Good potential for audience engagement	Some potential for engagement	Low engagement potential	Likely to be unengaging
Applicability	General applicability	Topic has wide relevance or practical significance beyond its niche	Some broader applicability or potential impact	Limited applicability or relevance	Relevance is very narrow or unclear	No broader relevance

### 2.3 Human reviewers

Reviewers of abstracts were directors and/or principal investigators at Nationwide Children's Hospital, representing diverse areas of expertise including child mental health, cardiovascular health, biopathology, and other fields. A total of 14 reviewers divided the task of grading 160 abstracts, with each reviewer evaluating a different number of abstracts based on their availability. Human reviewers were masked from AI-generated abstract scores and completed gradings independently. Each abstract was rated by two randomly assigned reviewers.

### 2.4 Large language models

We interfaced with ChatGPT-5, Gemini-3-Pro, and Claude-Sonnet-4.5 through API calls to grade abstracts based on the judging rubric. After a pilot test with 20 abstracts, we employed a batch processing strategy, sending 10 abstracts per API call to enable cross-comparison of abstracts within each batch, which facilitates the internal calibration of scores and reduces the total number of API requests. Compared to single-abstract evaluation, the batch grading produced systematically lower average criterion scores (Supplementary Table 4) while improving agreement between LLMs and human experts (Supplementary Table 5), suggesting that batch evaluation produces better-calibrated, more human-aligned scores. Abstract position within a batch had no significant effect on LLM scoring, with ANOVA revealing no significant differences across positions 1–10 for any criterion or LLM, indicating the absence of primacy or recency effects (Supplementary Table 6A). Randomizing abstracts to different batches, which changes the batch composition and comparison cohort, introduced either non-significant or significant but small mean score differences, which did not affect the overall findings (Supplementary Table 6B).

Through prompt engineering, we sent the following user prompt to each LLM agent at every API call. Importantly, the complete judging rubric, including all criteria and their behavioral anchors (Table 2), was embedded verbatim in the prompt as a text string, ensuring that the LLMs received the full evaluation framework for each scoring task. Detailed API parameters and access dates can be found in Supplementary Table 2.

The prompt began with the following instruction with the full rubric text inserted immediately afterward:

“You are a careful and responsible reviewer grading research abstracts for a conference. The abstracts start with a title and a 50-word description and include the following sections: importance, objective, methods/design, results/findings, conclusions. Here are seven criteria that must be included in the grading: Clarity and Organization, Research Question/Objective, Results/Expected Outcomes, Significance/Impact, General Applicability, Potential for Engagement, Overall Impression. Here are 10 abstracts. Please grade based on the provided rubric.”

### 2.5 Scoring patterns

We examined scoring patterns by comparing composite score distributions across LLM and human reviewers. We used boxplots to visualize the median, interquartile range, and outliers of composite scores for each rater.

### 2.6 Inter-rater reliability

We used intraclass correlation coefficient (ICC) to quantify inter-rater reliability. ICC decomposes the total variance into variance due to true differences between subjects vs. variance due to rater differences and measurement error; the higher the proportion attributable to true differences between subjects, the better the rater agreement (Bartko, 1966). ICC values range from 0 to 1, where 0 indicates no agreement and 1 indicates perfect agreement, with thresholds defined as < 0.2 poor, 0.2–0.4 fair, 0.4–0.6 moderate, 0.6–0.8 good, 0.8–1.0 excellent (Landis and Koch, 1977).

We calculated ICC for each criterion using two approaches. For agreement among the three LLM agents (ChatGPT-5, Gemini-3-Pro, and Claude-Sonnet-4.5), we used a two-way random effects model with absolute agreement definition and rater average unit, specified as ICC (2, k), as the same three LLMs evaluated all abstracts. For agreement between human reviewers and each individual LLM, we used a one-way random effects model to account for two random human reviewers grading each abstract, specified as ICC (1, k). Sensitivity power analyses for both types of ICC calculations, given the fixed sample constraints (*n* = 160, *k* = 3, α = 0.05, *1–*β = 0.80), revealed a minimum detectable ICC of 0.119, suggesting the study was adequately powered to detect ICC estimates above this value.

### 2.7 Visual agreement patterns

We constructed Bland-Altman plots (Bland and Altman, 1986) to visually inspect the agreement between human reviewers and each LLM on composite scores for 160 abstracts. For each comparison, we plotted the difference between LLM and mean human reviewer scores (y-axis) against the average of the two measurements (x-axis). The plots display the mean difference and 95% limits of agreement (±1.96 *SD*) to identify systematic bias and score-dependent variation in agreement.

## 3 Results

### 3.1 Scoring patterns

**Figure 1:**
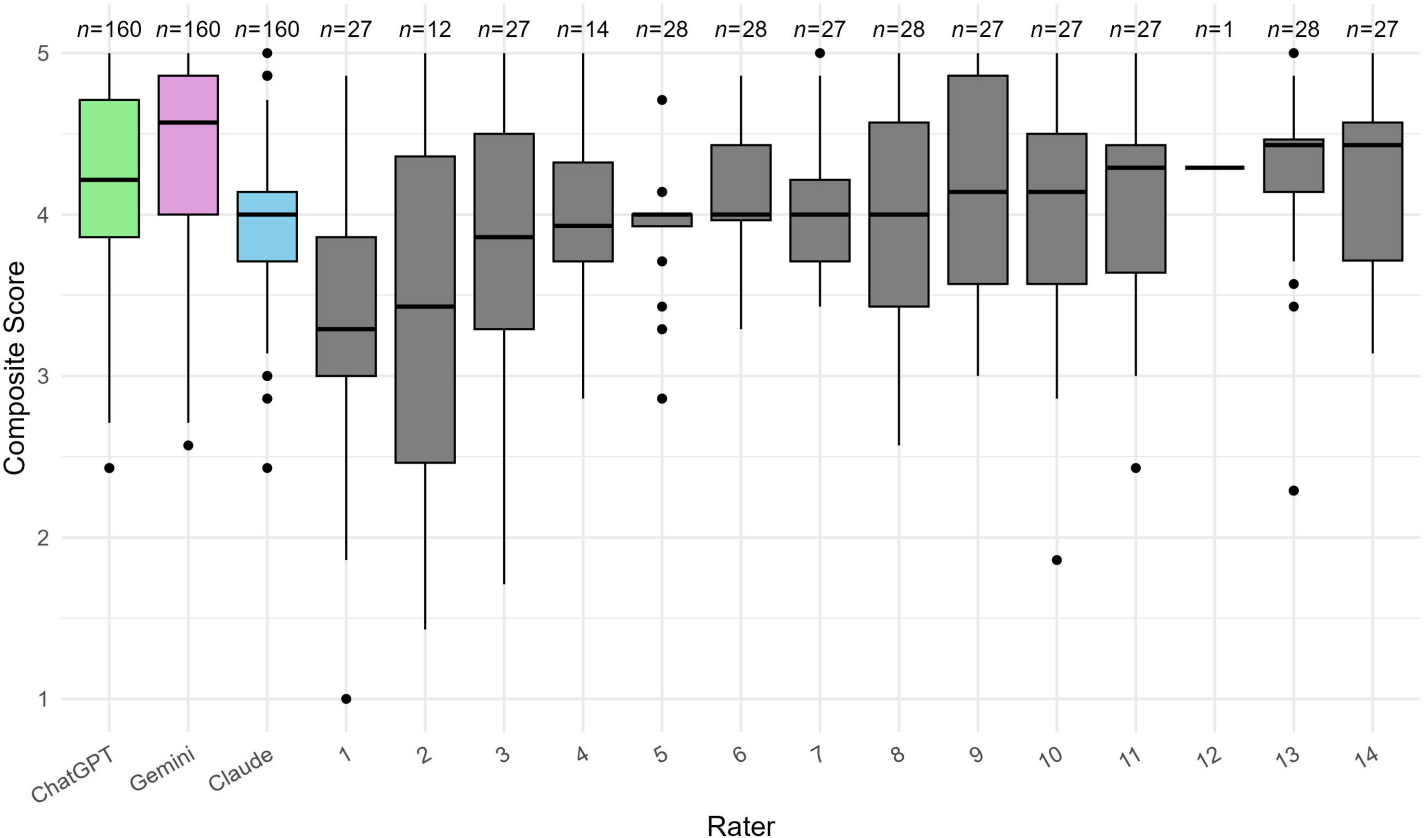
Boxplots of LLM and human reviewers' composite score distribution, averaging across all evaluation criteria, where LLMs showed more consistent scoring patterns than human reviewers.

The boxplots of composite scores (Figure 1) revealed distinct scoring patterns between LLM and human reviewers. The three LLMs, each scoring all 160 abstracts, maintained consistent scoring patterns characterized by narrow interquartile ranges (IQRs) and few outliers. In contrast, the 14 human reviewers, each grading between 1 and 28 abstracts, exhibited marked heterogeneity in their scoring behaviors, with substantial variations in IQRs, outlier frequencies, and median scores. Among LLM agents, Gemini exhibited lenient scoring with the highest median and widest IQR, while Claude emerged as the most stringent rater with the lowest median and tightest IQR. ChatGPT fell between these two extremes, showing moderate median and variability. In addition to boxplots, Supplementary Figure 1 presented histograms of three LLM's composite score distributions, and Supplementary Table 1 presented mean and standard deviation of all evaluation scores.

### 3.2 Inter-rater reliability comparing AI models to each other

**Figure 2:**
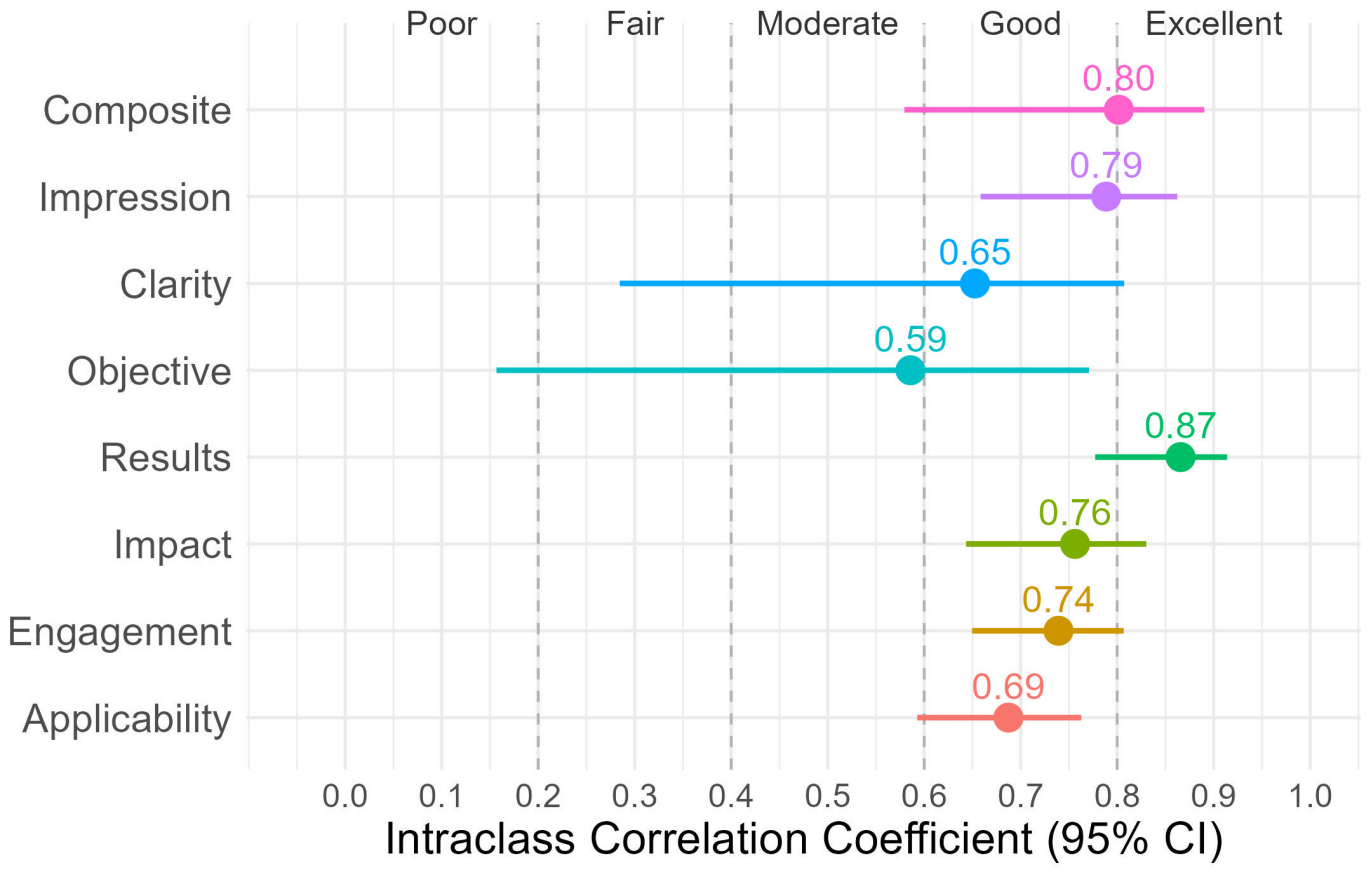
Intraclass Correlation Coefficient (ICC) with 95% Confidence Interval comparing ChatGPT, Gemini, and Claude across evaluation criteria, with good-to-excellent agreement observed for all criteria except objective.

ICC analysis between ChatGPT, Gemini, and Claude demonstrated promising inter-rater reliability across AI models (Figure 2). Two criteria achieved excellent agreement: composite scores [ICC (2, k) = 0.80] and results (0.87). Five criteria reached good agreement: impression (0.79), clarity (0.65), impact (0.76), engagement (0.74), and applicability (0.69). However, clarity showed a wider confidence interval spanning from fair to good, indicating greater uncertainty around this estimate. Objective (0.59) was the only criterion showing moderate agreement among the LLMs, and its lower bound of confidence extended into the poor range, suggesting high variability in how these models evaluate the significance of research questions. Overall, the good-to-excellent agreement across seven out of eight criteria indicates that the three LLMs apply evaluation standards consistently and similarly to each other, but they could have higher variability in certain criteria, which leads to less precise and stable estimates.

### 3.3 Inter-rater reliability comparing human reviewers and AI

**Figure 3:**
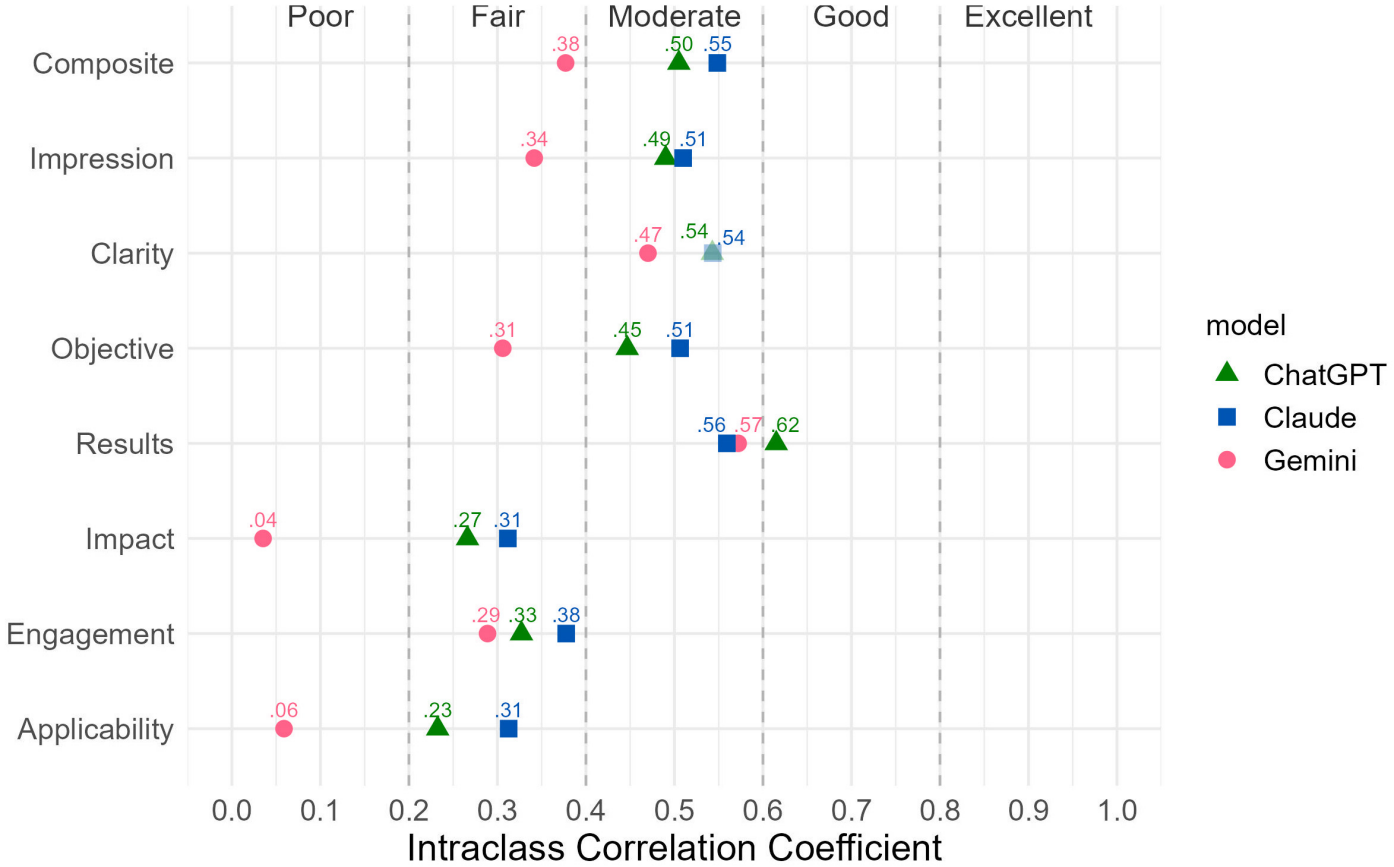
Intraclass Correlation Coefficients (ICCs) between human reviewers and individual LLM agent across evaluation criteria, where LLMs showed moderate agreement with humans on objective criteria and markedly worse agreement on subjective dimensions.

ICC analysis between human reviewers and individual LLM agent revealed mixed results (Figure 3). In contrast to the good-to-excellent agreement observed among LLM agents, humans and LLMs showed substantially lower inter-rater reliability, with each LLM agent exhibiting a distinct reliability profile.

ChatGPT demonstrated fair-to-moderate consistency with human scores: composite [ICC (1, k) = 0.50], impression (0.49), clarity (0.54), and objective (0.45) all showed moderate agreement, with results (0.62) achieving borderline good agreement. However, subjective dimensions showed weaker alignment, with impact (0.27), engagement (0.33), and applicability (0.23) demonstrating only fair agreement.

Gemini exhibited the weakest reliability with human reviewers across nearly all criteria: only clarity (0.47) and results (0.57) achieved moderate agreement, while composite (0.38), impression (0.34), objective (0.31), and engagement (0.29) all showed fair agreement. Particularly concerning, Gemini showed negligible and non-significant agreement with human reviewers on impact (0.04, *p* = 0.391) and applicability (0.06, *p* = 0.323). Gemini's mean scores for impact and applicability were 4.47 (*SD* = 0.56) and 3.92 (*SD* = 0.76; Supplementary Table 1), which were slightly higher than those of ChatGPT [4.33 (*SD* = 0.58); 3.86 (*SD* = 0.67)] and Claude [4.19 (*SD* = 0.55); 3.75 (*SD* = 0.61)] and with comparable variability. This suggests that the zero ICCs were not due to restricted score range but may reflect a systematic mismatch in how Gemini and human reviewers assessed these dimensions. Given the black-box nature of LLM transformer architectures, the exact mechanism underlying this misalignment remains unclear, and future research could explore whether this pattern stems from model training differences or rubric interpretation.

Claude demonstrated the strongest alignment with human reviewers across most criteria. Five criteria exhibited moderate agreement: composite (0.55), impression (0.51), clarity (0.54), objective (0.51), and results (0.56). The remaining three criteria showed fair agreement: impact (0.31), engagement (0.38), applicability (0.31). Claude matched ChatGPT's performance in evaluating abstract overall quality and content-specific aspects but proved to be more reliable when assessing subjective dimensions.

### 3.4 Visual agreement patterns

**Figure 4:**
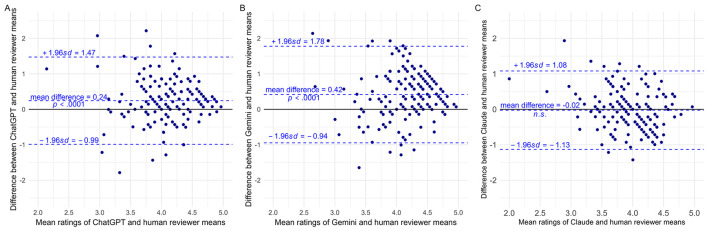
Bland-Altman plots of composite score differences between each LLM and human mean ratings. **(A)** ChatGPT-human comparison. **(B)** Gemini-human comparison. **(C)** Claude-human comparison. Three LLMs showed acceptable or negligible systematic differences from human mean ratings.

The Bland-Altman plots of composite scores visually validated the moderate agreement found between human reviewers and LLM, with additional information on systematic bias and agreement trend (Figure 4).

In Figure 4A, the Bland-Altman plot displays agreement between ChatGPT and human mean ratings. The mean difference of 0.24 (paired *t*-test, *p* < 0.0001) indicated a significant but small positive bias, with ChatGPT assigning slightly higher scores on average. About 95% of differences fall between −0.99 and 1.47, reflecting reasonable overall concordance between the two rating methods. Visual inspection of the plot suggested that differences approached zero as average scores increased, particularly for cases with mean ratings above 4.5. Spearman's rank correlation revealed a weak negative association between the absolute differences and mean ratings (Spearman's ρ = −0.16, *p* = 0.042), indicating slightly better agreement at higher scores. However, given the small effect size and marginal significance, this trend had limited practical impact and should be interpreted with caution.

Figure 4B displays the agreement between Gemini and human mean ratings. The two rating methods showed a mean difference of 0.42 (paired *t*-test, *p* < 0.0001), indicating a systematic positive bias of Gemini scores. The 95% limits of agreement ranged from −0.94 to 1.78, with most observations falling within these limits. There was a weak negative association between the differences and mean ratings (Spearman's ρ = −0.22, *p* < 0.01), suggesting that the agreement got slightly better when the scores increased. Similarly, this trend should be interpreted with caution due to the small effect size. Consistent with the fair reliability observed in the ICC analysis, Gemini exhibited the largest systematic bias and the widest range of disagreement among the three LLMs evaluated.

In Figure 4C, Claude demonstrated the strongest agreement with human raters, with a near-zero mean difference of −0.02 that was not statistically significant (paired *t*-test, *p* = 0.5771), corresponding to a negligible systematic bias between the two rating methods. The 95% limits of agreement ranged from −1.13 to 1.08, representing the narrowest range among the three LLMs. Visual inspection suggested a constant spread of differences across the full range of mean ratings, and Spearman's rank correlation confirmed no association between the absolute differences and the mean ratings (Spearman's ρ = 0.03, *p* = 0.69), indicating that Claude maintained stable agreement with human raters across all score levels.

### 3.5 Exploratory cost analysis

Three LLMs, respectively graded 160 abstracts within 10 min at a cost of less than $1. API pricing per million tokens varies across models at the access time of December 2025: for input tokens, ChatGPT-5 costs $1.25, Gemini-3-Pro $2, and Claude-Sonnet-4.5 $3; for output tokens, ChatGPT-5 costs $10, Gemini-3-Pro $12, and Claude-Sonnet-4.5 $15. At this small scale, the three models demonstrated comparable cost efficiency, with 160 abstracts requiring approximately 120,000 input tokens and 18,000 output tokens. It is worth noting that LLM API pricing has been declining rapidly with each new model generation, and the costs reported here could decrease by more than 50% within a 1-year span (LLM API Pricing Comparison, 2025). Aside from token prices, actual API costs scale with the number of abstracts, sophistication of prompts, and output detail level. Since costs accumulate on a per-token basis, longer prompts with detailed rubrics and extended qualitative feedback will increase expenses proportionally. API budget planning will become a crucial factor for deployment if LLM evaluation is used in large conferences with thousands of submissions. Generally speaking, using LLM to grade hundreds of scientific abstracts appears efficient and affordable for individuals and institutions with reviewing tasks.

## 4 Discussion

This study evaluated the feasibility of using large language models to review scientific research abstracts. Three LLMs—ChatGPT-5, Gemini-3-Pro, and Claude-Sonnet-4.5—showed good-to-excellent agreement with each other and moderate agreement with human experts on abstract overall quality and content-specific criteria, yet reliability deteriorated markedly on subjective dimensions. Bland-Altman plots confirmed the three models' reasonable concordance with human reviewers on abstract overall quality, as all three models showed acceptable or negligible systematic bias compared to human mean ratings. Collectively, these findings underscore the potential of LLMs to facilitate peer review, while their practical implementation in real-world settings needs further investigation.

LLMs had pragmatic advantages when serving as additional reviewers for abstract evaluation. First, they provide rapid reviews at a low cost. Human reviewers took over 2 weeks to complete ratings at considerable expense. A rough estimate suggests 3 min per abstract × 160 abstracts × 2 reviewers = 16 h of reviewer time, plus the opportunity cost of researchers spending time on reviews rather than research, and the fiscal cost of salary and benefits. Second, simply averaging this LLM review with other ratings will improve overall decision reliability, due to the Spearman-Brown “prophecy” formula (adding another judge of *k* items has the same effect on reliability as lengthening the test by *k* items) (Wiggins, 1973). Third, LLMs could evaluate abstract content independently without direct access to author demographics or prior field knowledge, enhancing their resistance to bias or criterion contamination (Sounderajah et al., 2025). Fourth, LLMs apply one rubric across entire abstract sets and provide uniformly calibrated ratings, mitigating the problem of between-rater variance (McGraw and Wong, 1996). When reviewers with different rating standards evaluate separate subsets of abstracts, some applicants are systematically advantaged or disadvantaged based on reviewer assignment rather than content quality. Adding an LLM reviewer thus improves both consistency and equity.

ChatGPT and Claude's moderate agreement with human reviewers confirms that AI-generated scores have reference values for overall quality and objective criteria. This suggests an alternative use case, where LLMs serve as an initial screening mechanism to filter out abstracts with clear deficiencies in structure and data reporting, thus reducing the overall reviewing workload. Such a two-stage review process resembles to triage or selection ratio models often used in admissions or hiring settings (Wiggins, 1973; Guion, 2011). Nevertheless, the score-dependent agreement patterns observed for ChatGPT (ρ = −0.16, *p* = 0.042) and Gemini (ρ = −0.22, *p* < 0.01), though with small effect sizes, suggest that LLM-human agreement may weaken at lower score ranges. One partial explanation for this visual pattern is an artifact of range restriction. In Bland–Altman plots, discrepancies are plotted against the average rating. When the average approaches the scale extremes (i.e., near the ceiling or floor), both raters are likely to assign similarly high or low scores, which mechanically constrains the possible discrepancy between them. Consequently, disagreement may appear smaller at the extremes and larger in the mid-range. Nevertheless, we recommend that practitioners examine LLM performance for quality-dependent biases prior to deployment and incorporate human judgements for abstracts filtering if such bias is detected. More importantly, LLMs' diminished performance in evaluating subjective criteria suggests that they function best as complementary tools in research evaluation, while human expertise remains essential for assessing the subjective dimensions of research quality. Thus, the “extra reviewer” deployment may be preferable to a triage model in settings where subjective criteria are paramount. Rather than replacing human experts, AI reviewers can augment their capabilities, freeing human resource to focus on abstracts requiring nuanced judgment, while enhancing consistency and objective aspects of the review process.

The good-to-excellent consistency between ChatGPT, Gemini, and Claude across evaluation criteria implies that popular LLMs, all built on the transformer structure, respond similarly when given identical user prompts and scientific abstracts. Nevertheless, the remaining variability in abstract scores demonstrates that LLM agents, each trained on distinct datasets and engineered with unique features, develop meaningfully different review styles, evident in their score distributions (Supplementary Figure 1). Claude's stronger alignment with human reviewers and Gemini's notably weaker performance underscore the importance of careful model selection when considering LLMs as tools for scientific abstract review. Conference committees could evaluate models based on their reviewing characteristics (lenient vs. stringent), agreement with human experts, and cost-effectiveness.

Limitations include that sample abstracts predominantly focused on pediatric health research, which does not represent the diversity of topics encountered at national or international conferences. The feasibility of LLM-assisted abstract review in larger-scale, more diverse conference settings, or their performance with humanities and physical science content, requires further investigation. Second, the human reviewers' expertise did not always align closely with the content of their randomly assigned abstracts, and all reviewers used a single “one size fits all” rubric. While peer review at specialized journals typically benefits from better content familiarity and expertise, generalist journals, grant review processes, and conferences with broad scope often encounter similarly wide variation in reviewer-content matching. Under these conditions, the human reviewers achieved only moderate inter-rater reliability on abstract overall quality and content-specific criteria, and poor-to-fair reliability on subjective dimensions [ICC (1, k) = 0.151–0.553; Supplementary Table 3]—consistent with metrics reported in meta-analyses (Bornmann et al., 2010; Reinhart, 2009)—constraining the upper bound of possible LLM-human concordance (Kraemer, 1992). Therefore, the agreement between human reviewers and LLMs should not be interpreted as a gold-standard benchmark for AI's research evaluation capabilities. Rather, this study offers preliminary insights into the potential applications of AI for abstract grading, with findings that warrant further investigation under more controlled conditions. Third, we only tested three popular AI models—ChatGPT-5, Gemini-3-Pro, and Claude-Sonnet-4.5—which cannot fully represent the variability of rapidly evolving AI models. Our findings should be interpreted cautiously when applied to other models with different architectures or specialized capabilities. Finally, the batch strategy of sending 10 abstracts per prompt and the assignment of human reviewers to abstract subsets introduced potential score interdependence, as scores may be influenced by the other abstracts evaluated together. This violates the strict independence assumption underlying ICC calculations. However, such interdependence is inherent to review process involving multiple submissions and is difficult to avoid in real-world contexts. Readers are encouraged to interpret ICC estimates in light of this constraint, with emphasis on overall agreement trends rather than exact coefficient values.

Susskind's distinction between process-oriented vs. outcome-oriented AI applications illuminates next steps for AI-assisted research evaluation (Susskind, 2025). Our team adopted a process-oriented approach, providing identical rubrics to AI and human reviewers and evaluating how closely LLM outputs matched human scores. To shift toward the outcome-oriented thinking advocated by Susskind, future work could employ substantially more detailed rubrics with 30 or 40 items that emphasize objective and quantitative criteria where LLMs excel. Such criteria may include grammatical correctness, structured abstract compliance, result concreteness (e.g., whether numerical outcomes are reported), methodological transparency (e.g., sample size and statistical approach are specified), and topical relevance to the conference scope. While such granularity would burden human reviewers, LLMs can consistently apply detailed frameworks at minimal additional cost, as demonstrated by prior works on systematic review and quality coding (Zhou and Hu, 2025; Li et al., 2024; Scherbakov et al., 2025). A hybrid workflow could be structured with LLMs first screening all submissions to flag abstracts that fail to meet basic reporting standards, followed by human reviewers evaluating shortlisted abstracts on subjective dimensions. The specific quality thresholds used to filter abstracts would depend on the target acceptance rate, human reviewer availability, and conference committee priorities. The psychometric benefits—improved reliability (Wiggins, 1973), broader content coverage (Guion, 2011), and consistent calibration—ultimately serve researchers' need for accurate, unbiased evaluation rather than simply more reviewers.

There are several additional directions for future research. First, future work should explore prompt variations to identify the most effective prompts for abstract evaluation tasks, as changes in prompt wording, structure, or context may meaningfully influence scoring consistency and agreement with human experts. Second, while our findings indicated score-dependent agreement patterns for two of the three LLMs examined, the small effect sizes warrant cautious interpretation. Future studies with larger samples and clearer quality stratification would be better positioned to implement subgroup ICC analyses and examine whether agreement patterns differ systematically across low-, medium-, and high-quality abstracts, which will have direct implications for LLM deployment in peer review.

In conclusion, this study compared popular LLMs with one another and with human reviewers on abstract evaluation by examining score distributions and inter-rater reliability. ChatGPT-5, Gemini-3-Pro, and Claude-Sonnet-4.5 demonstrated scoring efficiency and consistency, with ChatGPT and Claude achieving moderate agreement with human reviewers on overall quality and objective dimensions, but all models showed limited reliability in assessing subjective criteria. Based on these findings, LLMs could serve as complementary tools to augment, rather than replace, human expertise in research evaluation.

## Data Availability

The API script, analysis code, and sufficient data for replication are available upon request from the corresponding author.
