# Supplementary material for: Evaluating large language models for abstract evaluation tasks: an empirical study
Source: Front Res Metr Anal. 2026 May 4;11:1807672. doi: 10.3389/frma.2026.1807672 (PMC13180880; doi:10.3389/frma.2026.1807672)
Supplement: Supplementary file 1 [file Data_Sheet_1.DOCX]

| **Supplementary Table 1**. Mean and standard deviation of evaluation scores of human reviewers and three LLMs. | | | | |
| --- | --- | --- | --- | --- |
| **Criterion** | **Humans** | **ChatGPT** | **Gemini** | **Claude** |
| Composite | 3.95 (0.71) | 4.17 (0.54) | 4.32 (0.58) | 3.91 (0.47) |
| Impression | 3.66 (0.90) | 4.13 (0.71) | 4.35 (0.82) | 3.92 (0.61) |
| Clarity | 4.27 (0.82) | 4.29 (0.71) | 4.49 (0.79) | 3.85 (0.57) |
| Objective | 4.19 (0.94) | 4.59 (0.59) | 4.78 (0.43) | 4.17 (0.56) |
| Results | 4.16 (0.98) | 4.12 (0.89) | 4.31 (0.94) | 3.84 (0.75) |
| Impact | 4.00 (0.84) | 4.33 (0.58) | 4.47 (0.56) | 4.19 (0.55) |
| Engagement | 3.67 (0.87) | 3.85 (0.65) | 3.95 (0.80) | 3.67( 0.59) |
| Applicability | 3.70 (0.73) | 3.86 (0.67) | 3.92 (0.76) | 3.75 (0.61) |

| **Supplementary Table 2**. API parameters specified for each model. | | | |
| --- | --- | --- | --- |
| **Model** | **Version** | **Parameters** | **Date of API access** |
| ChatGPT | gpt-5 | temperature=1, top_p=1.0, max_tokens=4096 | 12/6/2025 |
| Gemini | gemini-3-pro-preview | temperature=1, top_p=0.95, top_k=64 | 12/6/2025 |
| Claude | claude-sonnet-4-20250514 | temperature=1, top_p=0.99, max_tokens=4096 | 12/6/2025 |

| **Supplementary Table 3**. ICC estimates and 95% CI of all comparisons, with *p*-value adjusted for False Discovery Rate (FDR) within each comparison | | | | |
| --- | --- | --- | --- | --- |
|  | **Between Humans** | | |  |
| Criteria | ICC(1,k) | 95% CI | *p*-value | FDR-adjusted *p* |
| Composite | 0.42 | 0.211-0.574 | <0.001 | <0.001 |
| Impression | 0.38 | 0.151-0.542 | <0.01 | <0.01 |
| Clarity | 0.55 | 0.391-0.672 | <0.001 | <0.001 |
| Objective | 0.44 | 0.233-0.586 | <0.001 | <0.001 |
| Results | 0.45 | 0.249-0.595 | <0.001 | <0.001 |
| Impact | 0.15 | -0.150-0.377 | 0.148 | 0.148 |
| Engagement | 0.28 | 0.018-0.471 | 0.019 | 0.025 |
| Applicability | 0.25 | -0.027-0.446 | 0.037 | 0.042 |
|  | **Between LLMs** | | |  |
| Criteria | ICC(2,k) | 95% CI | *p*-value | FDR-adjusted *p* |
| Composite | 0.80 | 0.658-0.862 | <0.001 | <0.001 |
| Impression | 0.79 | 0.658-0.862 | <0.001 | <0.001 |
| Clarity | 0.65 | 0.284-0.807 | <0.01 | 0.002 |
| Objective | 0.59 | 0.157-0.771 | <0.01 | 0.007 |
| Results | 0.87 | 0.777-0.914 | <0.001 | <0.001 |
| Impact | 0.76 | 0.643-0.830 | <0.001 | <0.001 |
| Engagement | 0.74 | 0.650-0.807 | <0.001 | <0.001 |
| Applicability | 0.69 | 0.593-0.763 | <0.001 | <0.001 |
|  | **ChatGPT vs. Humans** | | |  |
| Criteria | ICC(1,k) | 95% CI | *p*-value | FDR-adjusted *p* |
| Composite | 0.51 | 0.356-0.624 | <0.001 | <0.001 |
| Impression | 0.49 | 0.336-0.613 | <0.001 | <0.001 |
| Clarity | 0.54 | 0.405-0.653 | <0.001 | <0.001 |
| Objective | 0.45 | 0.280-0.580 | <0.001 | <0.001 |
| Results | 0.62 | 0.499-0.708 | <0.001 | <0.001 |
| Impact | 0.27 | 0.045-0.443 | 0.011 | 0.012 |
| Engagement | 0.33 | 0.124-0.489 | <0.01 | <0.01 |
| Applicability | 0.23 | 0.001-0.418 | 0.025 | 0.025 |

Supplementary Table 3 continued

|  | **Gemini vs. Humans** | | |  |
| --- | --- | --- | --- | --- |
| Criteria | ICC(1,k) | 95% CI | *p*-value | FDR-adjusted *p* |
| Composite | 0.38 | 0.189-0.527 | <0.001 | <0.001 |
| Impression | 0.34 | 0.143-0.500 | <0.001 | <0.01 |
| Clarity | 0.47 | 0.310-0.598 | <0.001 | <0.001 |
| Objective | 0.31 | 0.097-0.473 | <0.01 | <0.01 |
| Results | 0.57 | 0.443-0.675 | <0.001 | <0.001 |
| Impact | 0.04 | -0.256-0.268 | 0.391 | 0.391 |
| Engagement | 0.29 | 0.074-0.460 | <0.01 | <0.01 |
| Applicability | 0.06 | -0.225-0.286 | 0.323 | 0.369 |
|  | **Claude vs. Humans** | | |  |
| Criteria | ICC(1,k) | 95% CI | *p*-value | FDR-adjusted *p* |
| Composite | 0.55 | 0.412-0.657 | <0.001 | <0.001 |
| Impression | 0.51 | 0.362-0.628 | <0.001 | <0.001 |
| Clarity | 0.54 | 0.406-0.653 | <0.001 | <0.001 |
| Objective | 0.51 | 0.358-0.625 | <0.001 | <0.001 |
| Results | 0.56 | 0.426-0.665 | <0.001 | <0.001 |
| Impact | 0.31 | 0.104-0.478 | <0.01 | <0.01 |
| Engagement | 0.38 | 0.190-0.528 | <0.001 | <0.001 |
| Applicability | 0.31 | 0.105-0.478 | <0.01 | <0.01 |

| **Supplementary Table 4**. Differences between batch and single-abstract LLM scoring based on paired-sample t-test results. | | | |
| --- | --- | --- | --- |
| **Criterion** | **ChatGPT  Mean Dif** | **Gemini  Mean Dif** | **Claude  Mean Dif** |
| Composite | -0.01 | -0.39*** | -0.22*** |
| Impression | -0.03 | -0.48*** | -0.11** |
| Clarity | -0.02 | -0.38*** | -0.09* |
| Objective | -0.21*** | -0.23*** | -0.37*** |
| Results | -0.14** | -0.43*** | -0.13** |
| Impact | -0.14** | -0.47*** | -0.29*** |
| Engagement | -0.01 | -0.39*** | -0.29*** |
| Applicability | -0.48*** | -0.35*** | -0.24*** |

| **Supplementary Table 5**. ICC estimates and 95% CI of LLM-human accordance across evaluation criteria using single-abstract evaluation | | | | |
| --- | --- | --- | --- | --- |
|  | **ChatGPT vs. Humans** | | |  |
| Criteria | ICC(1,k) | 95% CI | *p*-value | FDR-adjusted *p* |
| Composite | 0.39 | 0.203-0.536 | <0.001 | <0.001 |
| Impression | 0.32 | 0.117-0.485 | <0.01 | <0.01 |
| Clarity | 0.41 | 0.228-0.550 | <0.001 | <0.001 |
| Objective | 0.23 | -0.006-0.413 | <0.05 | <0.001 |
| Results | 0.57 | 0.435-0.671 | <0.001 | <0.001 |
| Impact | 0.08 | -0.192-0.305 | 0.254 | 0.254 |
| Engagement | 0.23 | -0.001-0.416 | <0.05 | <0.05 |
| Applicability | 0.21 | -0.027-0.401 | <0.05 | <0.05 |
|  | **Gemini vs. Humans** | | |  |
| Criteria | ICC(1,k) | 95% CI | *p*-value | FDR-adjusted *p* |
| Composite | 0.07 | -0.214-0.292 | 0.300 | 0.480 |
| Impression | -0.10 | -0.432-0.165 | 0.751 | 0.858 |
| Clarity | 0.37 | 0.174-0.519 | <0.001 | <0.01 |
| Objective | 0.12 | -0.146-0.332 | 0.171 | 0.440 |
| Results | 0.46 | 0.292-0.587 | <0.001 | <0.001 |
| Impact | -0.43 | -0.861-0.085 | 0.994 | 0.994 |
| Engagement | 0.10 | -0.174-0.316 | 0.220 | 0.440 |
| Applicability | 0.04 | -0.250-0.271 | 0.378 | 0.505 |
|  | **Claude vs. Humans** | | |  |
| Criteria | ICC(1,k) | 95% CI | *p*-value | FDR-adjusted *p* |
| Composite | 0.52 | 0.370-0.633 | <0.001 | <0.001 |
| Impression | 0.47 | 0.306-0.596 | <0.001 | <0.001 |
| Clarity | 0.47 | 0.305-0.595 | <0.001 | <0.001 |
| Objective | 0.46 | 0.293-0.588 | <0.001 | <0.001 |
| Results | 0.59 | 0.460-0.685 | <0.001 | <0.001 |
| Impact | 0.33 | 0.127-0.491 | <0.01 | <0.01 |
| Engagement | 0.22 | -0.022-0.404 | <0.05 | <0.05 |
| Applicability | 0.30 | 0.082-0.465 | <0.01 | <0.01 |

| **Supplementary Table 6**. Examination of batch position effect on LLM scoring. Panel A. one-way ANOVA results testing whether abstract position within a batch (positions 1–10) affects LLM-assigned scores across eight evaluation criteria. Panel B. paired-sample t-test results examining between-batch cohort effects, comparing scores assigned to the same abstracts across different batch compositions. | | | |
| --- | --- | --- | --- |
| A. | | | |
| **Model** | ***F*-statistic range** | ***p*-value range** | **Conclusion** |
| ChatGPT | 0.372-1.404 | 0.191-0.947 | All *n.s.* |
| Gemini | 0.596-1.142 | 0.337-0.799 | All *n.s.* |
| Claude | 0.663-1.651 | 0.106-0.741 | All *n.s.* |
| Note. *df*=9 for all ANOVA comparisons. ANOVA revealed no significant effects across all criteria for any LLM | | | |
| B. | | | |
| **Criterion** | **ChatGPT  Mean Dif** | **Gemini  Mean Dif** | **Claude  Mean Dif** |
| Composite | -0.09** | 0.02 | 0.01 |
| Impression | -0.15** | 0.03 | 0.01 |
| Clarity | -0.10* | 0.09 | -0.04 |
| Objective | -0.10* | 0.05 | 0.01 |
| Results | -0.13* | 0.05 | 0.01 |
| Impact | -0.09 | 0.02 | 0.01 |
| Engagement | -0.06 | -0.04 | 0.04 |
| Applicability | -0.04 | -0.04 | 0.05 |
| Note. **p*<0.05, ***p*<0.01, all *p*-values adjusted for False Discovery Rate | | | |

**Supplementary Figure 1.**

Composite score histograms of human mean rating (A), ChatGPT (B), Gemini (C), and Claude (D)

A. B.

| 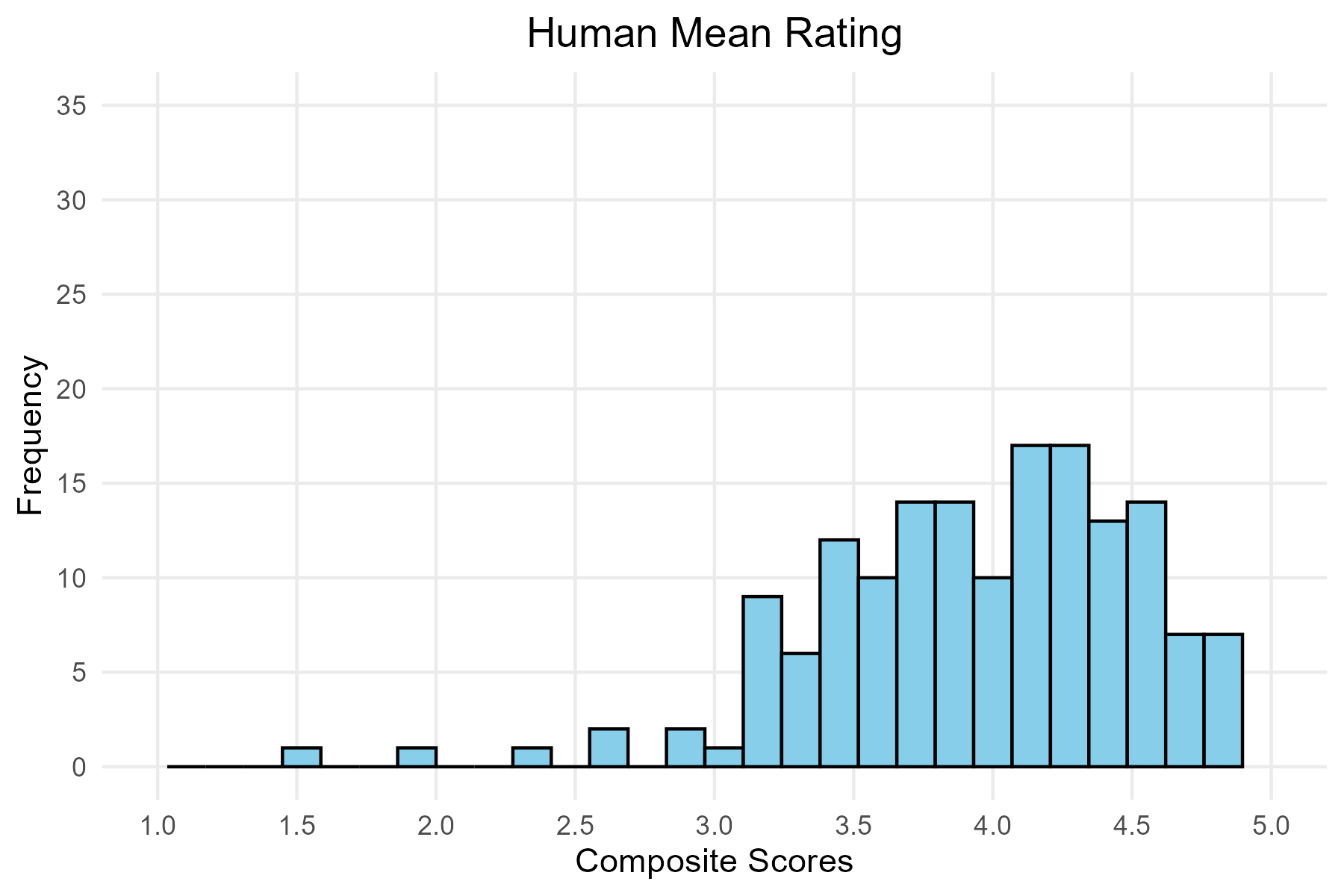  C.  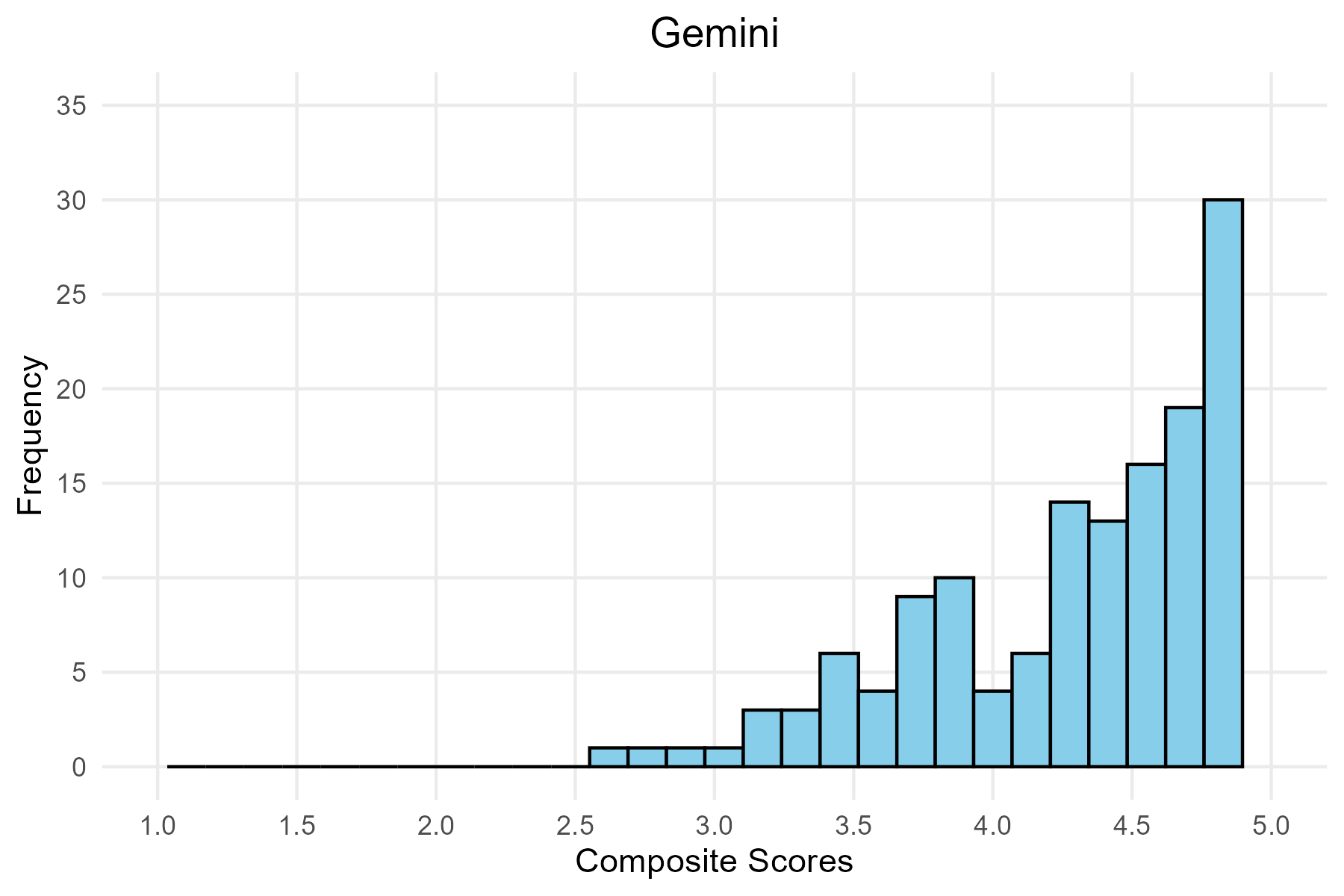 | 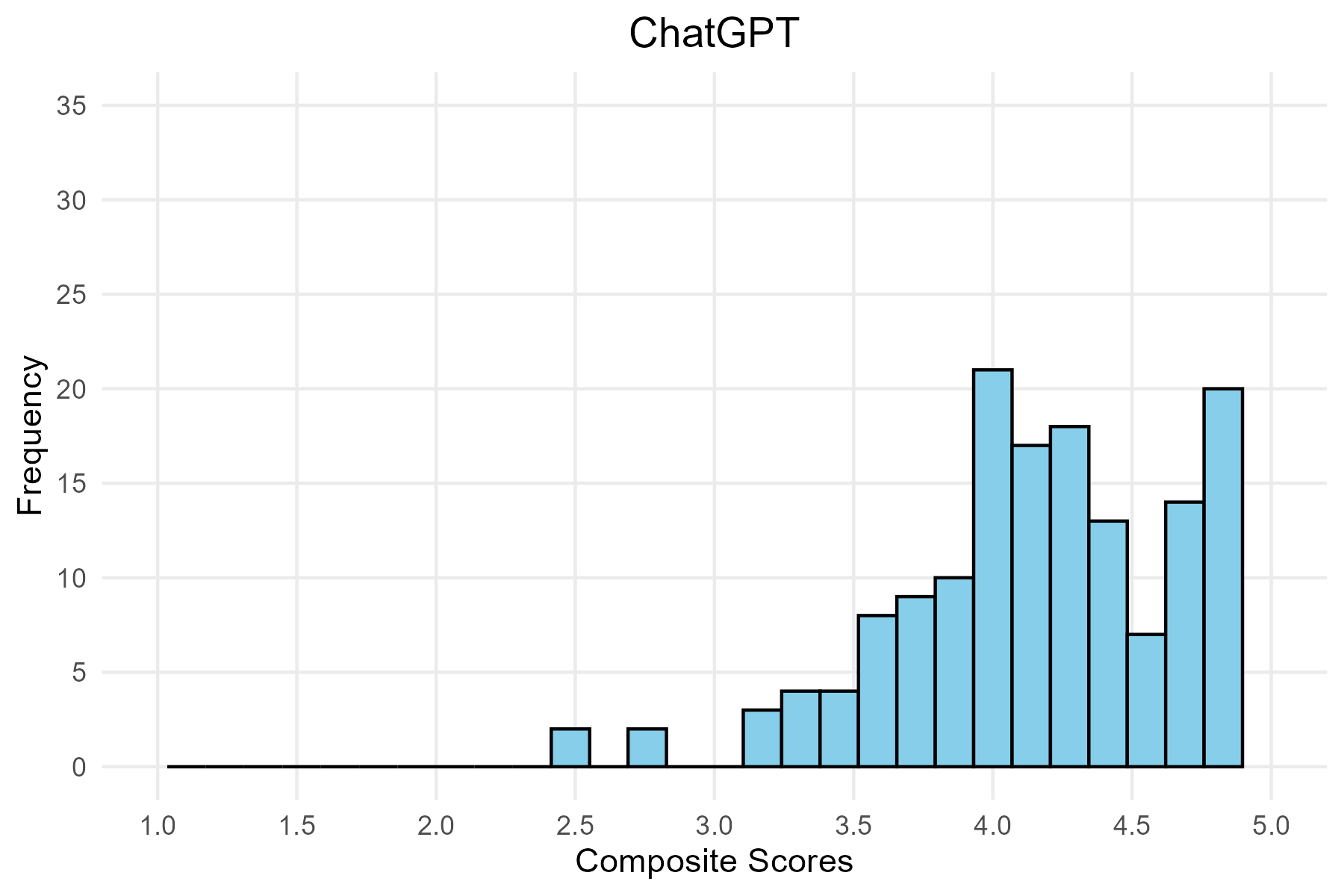  D.  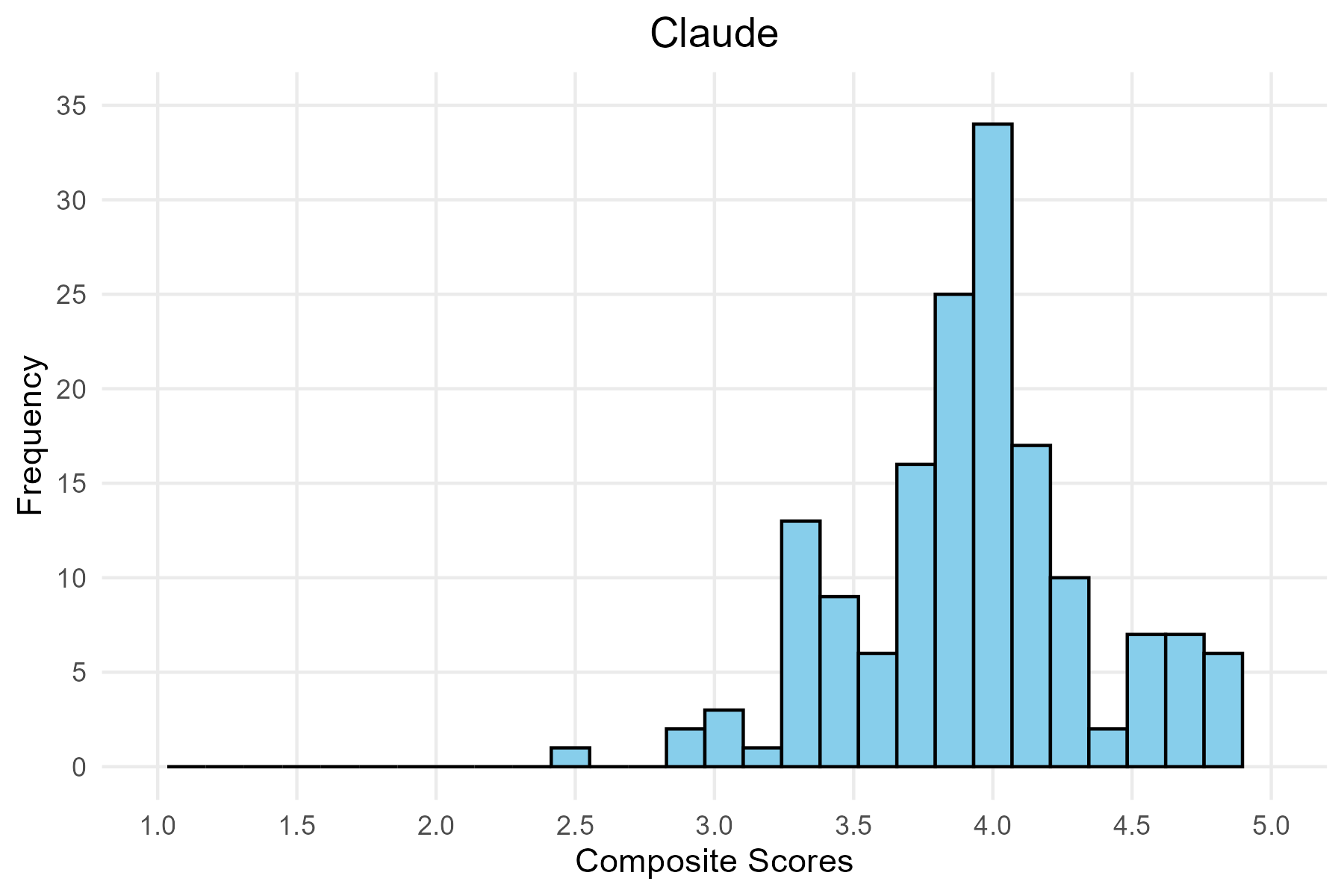 |
| --- | --- |
